# Calycosin alleviates allergic contact dermatitis by repairing epithelial tight junctions via down‐regulating HIF‐1α

**DOI:** 10.1111/jcmm.13763

**Published:** 2018-07-11

**Authors:** Zhirong Jia, Xiaotong Wang, Xiaoyu Wang, Pan Wei, Lianqu Li, Peng Wu, Min Hong

**Affiliations:** ^1^ Jiangsu Key Laboratory for Pharmacology and Safety Evaluation of Chinese Materia Medica School of Pharmacy Nanjing University of Chinese Medicine Nanjing China

**Keywords:** allergic contact dermatitis, calycosin, HIF‐1α, therapeutic targets, tight junctions

## Abstract

Calycosin, a bioactive component derived from Astragali Radix (AR; Huang Qi), has been shown to have an effect of anti‐allergic dermatitis with unknown mechanism. This study aims to investigate the mechanism of calycosin related to tight junctions (TJs) and HIF‐1α both in FITC‐induced mice allergic contact dermatitis and in IL‐1β stimulated HaCaT keratinocytes. Th2 cytokines (IL‐4, IL‐5 and IL‐13) were detected by ELISA. The epithelial TJ proteins (occludin, CLDN1 and ZO‐1), initiative key cytokines (TSLP and IL‐33) and HIF‐1α were assessed by Western blot, real‐time PCR, immunohistochemistry or immunofluorescence. Herein, we have demonstrated that allergic inflammation and the Th2 cytokines in ACD mice were reduced significantly by calycosin treatment. Meanwhile, calycosin obviously decreased the expression of HIF‐1α and repaired TJs both in vivo and in vitro. In HaCaT keratinocytes, we noted that IL‐1β induced the deterioration of TJs, as well as the increased levels of TSLP and IL‐33, which could be reversed by silencing HIF‐1α. In addition, administration of 2‐methoxyestradiolin (2‐ME), a HIF‐1α inhibitor,significantly repaired the TJs and alleviated the allergic inflammation in vivo. Furthermore, TJs were destroyed by DMOG or by overexpressing HIF‐1α in HaCaT keratinocytes, and simultaneously, calycosin down‐regulated the expression of HIF‐1α and repaired the TJs in this process. These results revealed that calycosin may act as a potential anti‐allergy and barrier‐repair agent via regulating HIF‐1α in AD and suggested that HIF‐1α and TJs might be possible therapy targets for allergic dermatitis.

## 1 INTRODUCTION

Allergic dermatitis (AD) is an common chronic or relapsing inflammatory skin disease that often precedes asthma and allergic disorders.^1^ The clinical picture of AD is typically characterized by alternate periods of remissions and exacerbations, while it can also be persistent.^2^ Topical corticosteroids (TCS) are considered the mainstay of pharmacological treatment and the first‐choice drugs for AD therapy.^3^ However, the long‐term use of TCS, especially high potency, may cause local side effects. In rare cases, systemic effects may occur, more frequently in children.^4^ In addition, inadequate applications of TCS limit its ability to control dermatitis and fail to alleviate the recurrence of the disease.^5^, ^6^ Therefore, it is necessary to explore and develop more appropriate drugs for the better control of AD.

Calycosin, an isoflavonoid phytoestrogen isolated from Astragali Radix, was reported to possess antitumour and anti‐inflammation properties.^7^ The dried roots of Astragali Radix, called “Huang Qi” in Chinese, are well known as a basic traditional Chinese medicine for thousands of years. “Huang Qi” has powerful immunomodulatory function and is widely used for treating dermatitis, chronic rhinitis, asthma and glomerulonephritis.^8^, ^9^, ^10^ Calycosin‐7‐O‐β‐D‐glucoside and calycosin are the main active flavone of “Huang Qi,” and the former would transform into the latter in vivo.^11^ Our previous study showed that administration of calycosin in the initial stage of sensitization when the ear has not yet produced inflammation significantly reduced the ear inflammation in mice ACD model.^12^ However, the impact of calycosin treated throughout the ACD model when a large number of inflammatory factors appear is unknown, and the mechanism is also worth exploring.

The epidermis of allergic dermatitis patients could have significant barrier disruption.^13^ Epidermal barrier dysfunction is a prerequisite for the penetration of high molecular weight allergens, such as pollens, house‐dust mite products, microbes and food.^14^, ^15^ A disturbed barrier alone could drive dendritic cells to enhance Th2 polarization in patients with AD through elevated antigen permeation.^16^ In recent years, more and more researches have demonstrated that the dysregulation of the epithelial barrier might enhance susceptibility and result in the allergen uptake, which initiates an allergic immune response by promoting the release of interleukin (IL)‐25, IL‐33 and thymic stromal lymphopoietin (TSLP),^17^ which are key initiators of allergic diseases leading to the induction and maintenance of Th2 responses.^18^, ^19^ The impairment of epidermal tight junctions can induce immune dysregulation in allergic dermatitis,^20^ and disruption of tight junctions was demonstrated by down‐regulation of a variety of proteins including CLDN1, occludin and ZO‐1. CLDN1 regulates the pathogenesis, severity and natural course of human AD, and reduced levels of CLDN1 in AD skin have been inversely correlated with expression of Th2 markers and a propensity to infection.^21^, ^22^, ^23^ It is noteworthy that restoring the expression of CLDN1 alleviates atopic dermatitis.^24^ So regulating tight junctions might involve in the mechanism of attenuation of AD.

AD is characterized by an increase in the release of local inflammatory factors, such as IL‐1β, TNF‐α and Th2 cytokines.^25^, ^26^ Many studies have shown that inflammatory cytokines play a vital role in the dysregulation of the epithelial barrier function.^27^, ^28^ HIF‐1α can be activated by these inflammatory mediators and can activate inflammation‐modulating proteins itself.^29^ Therefore, HIF‐1α may relate to the regulation of the epithelial barrier function. Furthermore, the role of HIF‐1α in inflammatory reactions associated with dermatitis has recently become evident.^30^ It is reported that UVB exposure may increase TSLP expression in keratinocytes through a HIF‐1α‐dependent mechanism.^31^ Moreover, a murine study has demonstrated that HIF‐1α protein is an early responder to UV radiation in the skin.^32^, ^33^ HIF‐1α may be an important pathway involved in processes such as delivery of “second signals” in contact hypersensitivity reactions to allergen.^34^ Although some plant isoflavones were reported to exert inhibition effect on HIF‐1α, the effect of calycosin on it in the context of allergic inflammation remains to be illustrated. Therefore, HIF‐1α is likely to play a role in AD and the anti‐allergy mechanism of calycosin may relate to it.

## 2 MATERIALS AND METHODS

### 2.1 Cell lines

HaCaT keratinocytes derived from ATCC (Manassas, VA, USA) were obtained from the National Infrastructure of Cell Line Resource (Beijing, China). HaCaT cells were cultured in modified Eagle's medium (MEM) supplemented with 10% foetal bovine serum (FBS, Gibco) and penicillin/streptomycin (BioSharp) in a 5% CO_2_ incubator (Thermo).

### 2.2 Animal

BALB/c mice were purchased from Shanghai Slack Laboratory Animal Co., Ltd. All animals were housed in a specific pathogen‐free facility at 18‐25°C and 40%‐70% humidity and were used at 6‐8 weeks of age. All procedures involving animals were approved by the Animal Care and Use Committee of Nanjing University of Chinese Medicine and performed strictly according to the Guide for the Care and Use of Laboratory.

### 2.3 Reagents and chemicals

The reagents used in this study were purchased as follows. Primers were ordered from GenScript (Nanjing, China). Calycosin was purchased from Tianjin Vientiane Hengyuan Technology Co., Ltd (China, Purity: ≥99%). Dexamethasone was a product from Tianyao Pharmaceutical Co., Ltd (Hubei, China). 2‐ME and DMOG were obtained from Selleck (Shanghai, China). LPS (lipopolysaccharides) were purchased from Sigma (Sigma‐Aldrich China, Shanghai, China). TNF‐α and IL‐1β were obtained from PeproTech (New Jersey, USA).

### 2.4 Mouse Th2‐mediated ACD model

After acclimatization for 3 days, the abdomens of BALB/c mice were shaved with a razor over an area of about 3 × 3 cm^2^. The abdominal skin of the mice was treated with 1.5% FITC (Sigma, St. Louis, MO, USA) in 80 μL of acetone and dibutylphthalate (1:1; vehicle) on days 1 and 2, and ears were treated with 20 μL of 0.6% FITC solution on day 6. On day 7 (24 hours after elicitation), ear thickness was measured with a thickness gauge (7301; Mitutoyo, Kawasaki, Japan), and ear swelling was calculated (ear thickness in each group minus the average ear thickness in the control group). The mice were then killed, and ear tissues were collected. The levels of IL‐4, IL‐5 and IL‐13 in ear tissue homogenate were detected by ELISA. Histopathological changes of the ears were determined by HE staining.

### 2.5 Drug administration

A: The dose‐response study of calycosin in ACD model

The establishment of FITC‐induced ACD model is as described above. In ACD model, mice were treated daily with calycosin (2, 10 or 50 mg/kg, intraperitoneally), DEX (0.67 mg/kg, intraperitoneally) or normal saline (model group) for 7 days during the whole course of the experiment. In this experiment, the ID50 was calculated by combining the results of ear swelling, lymphocyte infiltration and cytokines. The calculation process is as follows: calculate the inhibition rate (IR) of each indicator for each dose (2, 10 and 50 mg/kg) separately, and then, the IR was multiplied by their respective weight coefficients (ie IR of ear swelling*0.4, IR of lymphocyte infiltration*0.3 and IR of cytokines*0.3) and sum the 3 indicators to get the total inhibition rate and, finally, calculate ID50 by ID50 calculator software based on the total inhibition rate of each dose.

B: The effect investigation of 2‐ME on ACD.

The establishment of FITC‐induced ACD model is as described above. Mice were treated once daily with 2‐ME (50 mg/kg, intraperitoneally) in the whole course of ACD model.

C: The topical application of calycosin in ACD model

For topical application of calycosin, a kind of grease ointment matrix was made: put 0.05 g calycosin in a mortar, add 0.2 g of liquid paraffin, grind it into a paste, add 0.75 g of Vaseline in several portions and mix them. Mice ears were painted once daily with 1 mg of calycosin for 7 days during the whole course of the experiment.

D: The study of calycosin administered only in the initial stage of ACD model

The establishment of FITC‐induced ACD model is as described above. Mice were administered once daily with calycosin (50 mg/kg, intraperitoneally), DEX (0.67 mg/kg, intraperitoneally) or normal saline (model group) 1 day before treatment with FITC until day 3 of the model.

### 2.6 Western blotting

Western blotting was performed to assess the changes in the protein expression levels of HIF‐1α, CLDN1, occludin, ZO‐1, TSLP and IL‐33. HaCaT cells were homogenized in assay lysis buffer (RIPA: phenylmethylsulfonyl fluoride = 100:1). The BCA protein assay kit (Thermo Scientific) was used to quantify the amount of extracted protein. Protein samples (30 μg) were loaded on 10% SDS‐PAGE and transferred to polyvinylidene difluoride membranes (Millipore, Darmstadt, Germany). After blocking with 10% skim milk, the membranes were washed with Tris‐buffered saline (TBS), containing 0.1% Tween 20 (TBST) at room temperature. Primary antibodies against HIF‐1α, CLDN1 (1:1000 dilution; Abcam, Cambridge, MA, USA), ZO‐1, occludin, lamin A/C (1:1000 dilution; Proteintech Group, Wuhan, Hubei, China), TSLP, IL‐33 (1:1000 dilution, Santa Cruz Biotechnology, CA, USA) and GAPDH (Bioss, Beijing, China) were incubated at 4°C overnight. After washing, membranes were incubated at room temperature with the HRP‐conjugated goat anti‐rabbit IgG secondary antibody (1:10 000 dilution; AbSci, MD, USA) for 1 hour. After washing, the electrochemical luminescent substrates (Millipore, MA, USA) were used according to the manufacturer's protocol to visualize the proteins of interesting in the Bio‐Rad imaging system. Quantitative analysis of Western blot data was performed with ChemiScope analysis software, and values were normalized against respective GAPDH expressions.

### 2.7 Histological and immunohistochemical analysis

Ears were fixed in 10% neutral formalin, embedded in paraffin and sectioned. Dry tissue sections of 5 μm thickness at 60°C constant temperature box were baked for 30 minutes. Slides were undergone dewaxing, and hydration was performed with sequential dimethylbenzene soak for 20 minutes. Sequential ethanol soaks were carried out for 3 minutes, each starting 100% ethanol for twice, followed by 95%, 80% and 75% ethanol, and finishing with a 50% ethanol. Antigen was retrieved by citric acid buffer water bath heating by microwave oven at high fire for 12 minutes, followed by adding the buffer twice at high fire for 5 minutes every time and then restored at room temperature. Block endogenous peroxidase by incubating 10 minutes in 3% H_2_O_2_. Block non‐specific binding sites with 10% normal goat serum for 1 hour. Sections were used for haematoxylin and eosin (H&E) and immunohistochemical examination. For immunohistochemistry, the sections were incubated with primary antibody CLDN1, occludin and ZO‐1(1:100, 4°C, overnight), followed by incubation with HRP‐conjugated goat anti‐rabbit IgG secondary antibody according to the kit instructions (ZSGB‐BIO, Beijing, China). Peroxidase conjugates were subsequently visualized by utilizing diaminobenzidine (DAB) solution. The sections were then counterstained with haematoxylin and mounted on a coverslip. Between each step, the cells were extensively rinsed 3 times for 5 minutes each time. Staining was photographed by Olympus microscope (Mantra, PerkinElmer).

### 2.8 Immunofluorescence analysis

The changes in the expressions and distribution of CLDN1, occludin and ZO‐1 were analysed by immunofluorescence analysis. HaCaT cells were washed by PBS for 3 times and then fixed in ice methanol for 5 minutes at −20°C and washed 3 times with PBS. Blocking was performed for 1 hr at room temperature with 10% normal goat serum in phosphate‐buffered saline. Junction proteins were detected with the use of rabbit monoclonal antibodies against occludin, ZO‐1 and CLDN1 (1:100, 4°C, overnight), followed by incubation with the goat anti‐rabbit IgG‐FITC (1:200, room temperature, 1 hour, Abcam). Afterwards, the nucleus was stained with DAPI solution (1:2000, Beyotime) for 10 minute at room temperature. Between each step, the cells were extensively rinsed 3 times for 3 minutes each time. Staining was assessed with a live cell work station microscope (Carl Zeiss Jena).

### 2.9 Transfection with siRNA for HIF‐1α in HaCaT

In all experiments, 150 pmol siRNA (the target sequence of HIF‐1α‐specific siRNA: 5′‐GGCGAAGUAAAGAAUCUGATT‐3′, TranSheep Bio, Shanghai, China) were used to transfect 40%‐50% confluent cells according to the manufacturer's instructions. The Lipofectamine 2000 reagent (Life Technologies, Carlsbad, USA)was used to deliver siRNA into HaCaT cells growing in serum‐free opti‐MEM media. After 6 hours, the medium containing the siRNA‐lipid complexes was replaced with MEM containing 10% FBS. After transfection for 48 hours, the cells were stimulated with IL‐1β, and then, subsequent experiments were completed.

### 2.10 The transfection with pLenti‐CMV‐HIF‐1α in HaCaT cells

HaCaT cells were transiently transfected with pLenti‐CMV‐HIF‐1α (Asia‐Vector Biotechnology Co. LTD, Shanghai, China) for the overexpression of HIF‐1α. The human HIF‐1α was amplified by the following set of primers: 5′‐CGCAAATGGGCGGTAGGCGTG‐3′ and 5′‐TACGGGAAGCAATAGCATGA ‐3′ and packed into lentiviral vector pLenti‐CMV. The transfections were performed according to the manufacturer's instructions: 5 μg of pLenti‐CMV‐HIF‐1α or empty vector was used to transfect 70%‐80% confluent cells. Five μL of Lipofectamine 2000 reagent was used to deliver plasmid DNAs into HaCaT cells growing in serum‐free opti‐MEM media. After 6 hours, the medium was replaced with MEM containing 10% FBS. And subsequent experiments were completed 24 hours after transfection.

### 2.11 RNA isolation and quantitative real‐time PCR

Total RNA was extracted by 1 mL TRIZOL reagent per sample according to the manufacturer's instructions (Transgene). The RNA was first reverse‐transcribed into cDNA by a PrimeScript RT reagent Kit (Vazyme) and then subjected to qPCR with SYBR Green Master Mix (Vazyme). Real‐time PCR was performed on an Applied Biosystems 7500 real‐time PCR system. The cycle time value of the interested gene was normalized with GAPDH of the same sample; fold induction of gene expression was calculated by the ΔΔCt method. Results obtained from each PCR were pooled and statistically analysed. The following primers were used: HIF‐1α, 5′‐GCCGAGGAAGAACTATGA‐3′, 5′‐ACTGAGGTTGGTTACTGTT‐3′; TNF‐α, 5′‐GAGCACTGAAAGCATGATC‐3′, 5′‐GAAGAGGCTGAGGAACAA‐3′; IL‐1β, 5′‐AGGATATGGAGCAACAAGT‐3′, 5′‐GCAGGACAGGTACAGATT‐3′; TSLP, 5′‐AGAGCCTAACCTTCAATCC‐3′, 5′‐TTTATCTGAGTTTCCGAATAGC‐3′; IL‐33, 5′‐TGACGGTGTTGATGGTAA‐3′, 5′‐AGAGTGTTCCTTGTTGTTG‐3′; IL‐25, 5′‐CTCTACCACAACCAGACT‐3′, 5′‐CACACAAGCTAAGGAAACA‐3′; occludin, 5′‐CCATTAACTTCGCCTGTG‐3′, 5′‐TTGACCTTCCTGCTCTTC‐3′; CLDN1, 5′‐AATCTGAGCAGCACATTG‐3′, 5′‐GTCTTCCAAGCACTTCATAC‐3′; ZO‐1, 5′‐GATGGTGCTACAAGTGATG‐3′, 5′‐TCCGTGCTATACATTGAGT‐3′; GAPDH, 5′‐CTTCTTTTGCGTCGCCAGCCGA‐3′, 5′‐ACCAGGCGCCCAATACGACCAA‐3′.

### 2.12 Enzyme‐linked immunosorbent assay (ELISA)

Ears were ground into homogenates with ice‐phosphate‐buffered saline (PBS), and the homogenates were centrifuged at 3500 *g* at 4°C for 10 minutes. ELISAs were performed to relatively quantify protein in the ear tissue homogenate. All of the procedures followed the manufacturer's instructions. Mouse TNF‐α, IL‐1β, IL‐4,IL‐5 and IL‐13 ELISA kit were obtained from eBioscience Co., Ltd (San Diego, CA, USA).

### 2.13 Statistical analysis

Data are expressed as mean values + SD. Unpaired Student's *t* test was used when comparing 2 groups. One‐way ANOVA was used to compare multiple groups and 2 groups with Dunnett's test. Statistical analysis was performed by Prism 5.00 software (GraphPad, San Diego, CA, USA). The differences were considered significant for *P *<* *.05.

## 3 RESULTS

### 3.1 Calycosin inhibited allergic inflammation in FITC‐induced mice ACD model

**Figure 1:**
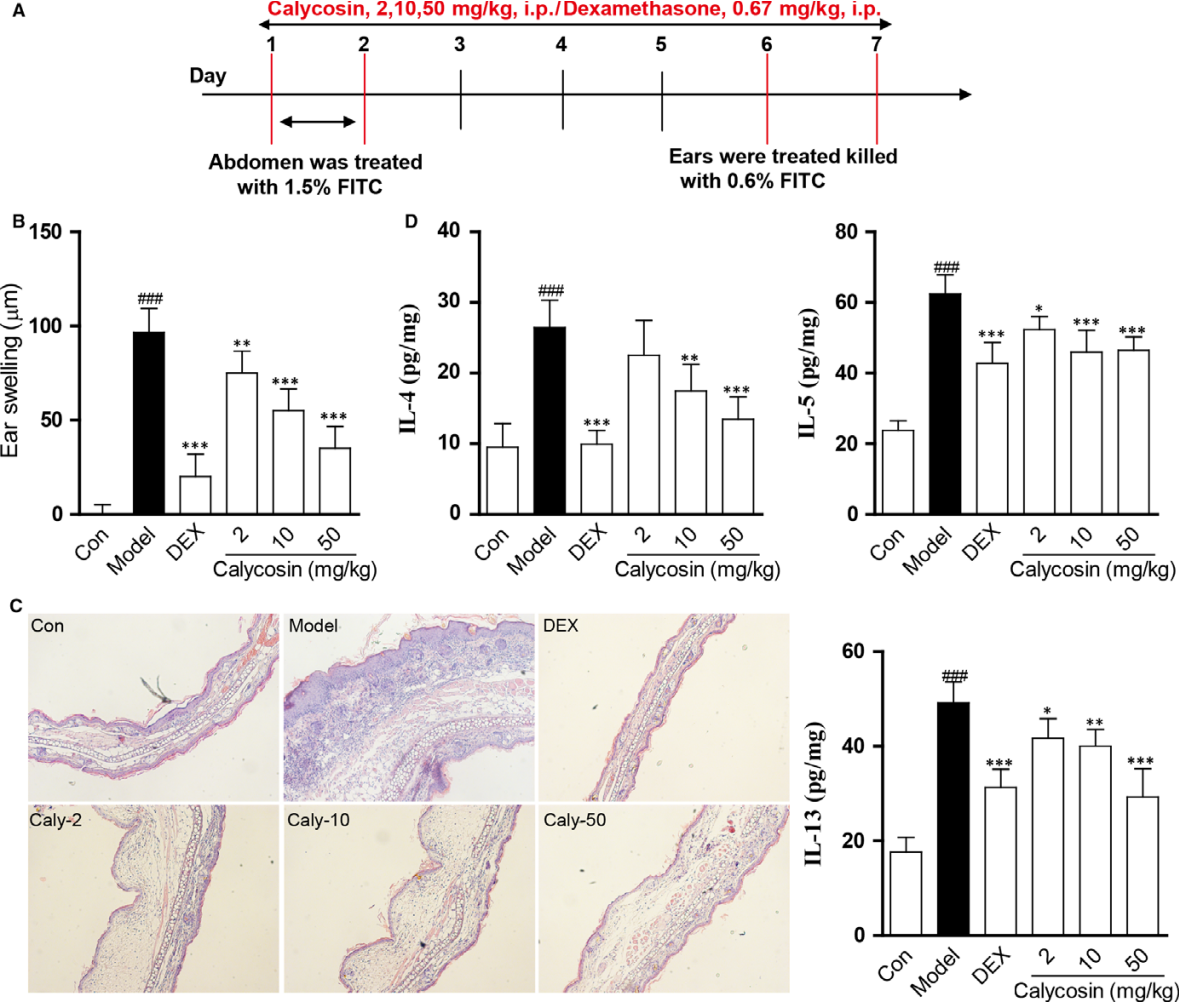
Calycosin inhibited FITC‐induced ACD‐associated allergic inflammation. A, Flow charts of the FITC‐induced ACD model. In ACD model, mice were treated daily with calycosin (2, 10 or 50 mg/kg, intraperitoneally), DEX (0.67 mg/kg, intraperitoneally) or normal saline (model group) for 7 days during the whole course of the experiment. B, Ear swelling was calculated on day 7 in the ACD model mice (means + SD, n = 6, ^###^ *P *<* *.001 vs control; ***P <* *.001, **P <* *.01 vs model). C, Haematoxylin and eosin (H&E)‐stained ear skin sections from FITC‐induced ACD mice model (n = 5; magnification: ×200). D, Calycosin reduced the levels of cytokines IL‐4, IL‐5 and IL‐13 in the ear tissue homogenates of the mouse model of ACD (mean + SD, n = 6, ^###^ *P *<* *.001 vs control; ***P <* *.001, **P <* *.01, *P <* *0.05 vs model)

To evaluate the activity of calycosin against ACD, a dose‐response study was carried out. In ACD model, mice were treated daily with calycosin (2, 10 and 50 mg/kg), DEX (0.67 mg/kg) or normal saline (model group) for 7 days during the course of the experiment. Ear tissues were collected 24 hours after the final FITC challenge (Figure 1A). It showed that the ear swelling, inflammatory cell infiltration and oedema were significantly inhibited by calycosin in a dose‐dependent manner (Figure 1B,C). Moreover, the levels of IL‐4, IL‐5 and IL‐13 in the ear tissues were also obviously inhibited by calycosin treatment (Figure 1D). In addition, the total inhibition rate of each dose (2, 10 and 50 mg/kg) is, respectively, 28.4%, 41.0% and 58.0%, and the ID50 was calculated to be 22.95 mg/kg. These results indicated that calycosin inhibits allergic inflammation in a dose‐dependent manner in mice Th2 ACD model.

Furthermore, we tried the topical application of calycosin in the whole course of ACD model. One mg of calycosin was painted to the ears once daily (Figure S1A). The results showed that ear swelling, the levels of IL‐4, IL‐5 and IL‐13 in the ear tissues and inflammatory infiltration were all significantly reduced by calycosin (Figure S1B‐D). Meanwhile, the effect of administration of calycosin only in the initial stage of ACD model was also investigated. Mice were administered once daily with calycosin (50 mg/kg, intraperitoneally), DEX (0.67 mg/kg, intraperitoneally) or normal saline (model group) 1 day before treatment with FITC until day 3 of the model (Figure S2A). The results showed that calycosin still inhibits the allergic inflammation significantly by decreasing the ear swelling, the levels of IL‐4, IL‐5 and IL‐13 in ear tissues, as well as the inflammatory infiltration (Figure S2B‐D). These results suggested that calycosin treated either in afferent phase or in the whole course exhibits well activity against ACD.

### 3.2 Epithelial tight junctions were repaired by calycosin in ACD mice model

**Figure 2:**
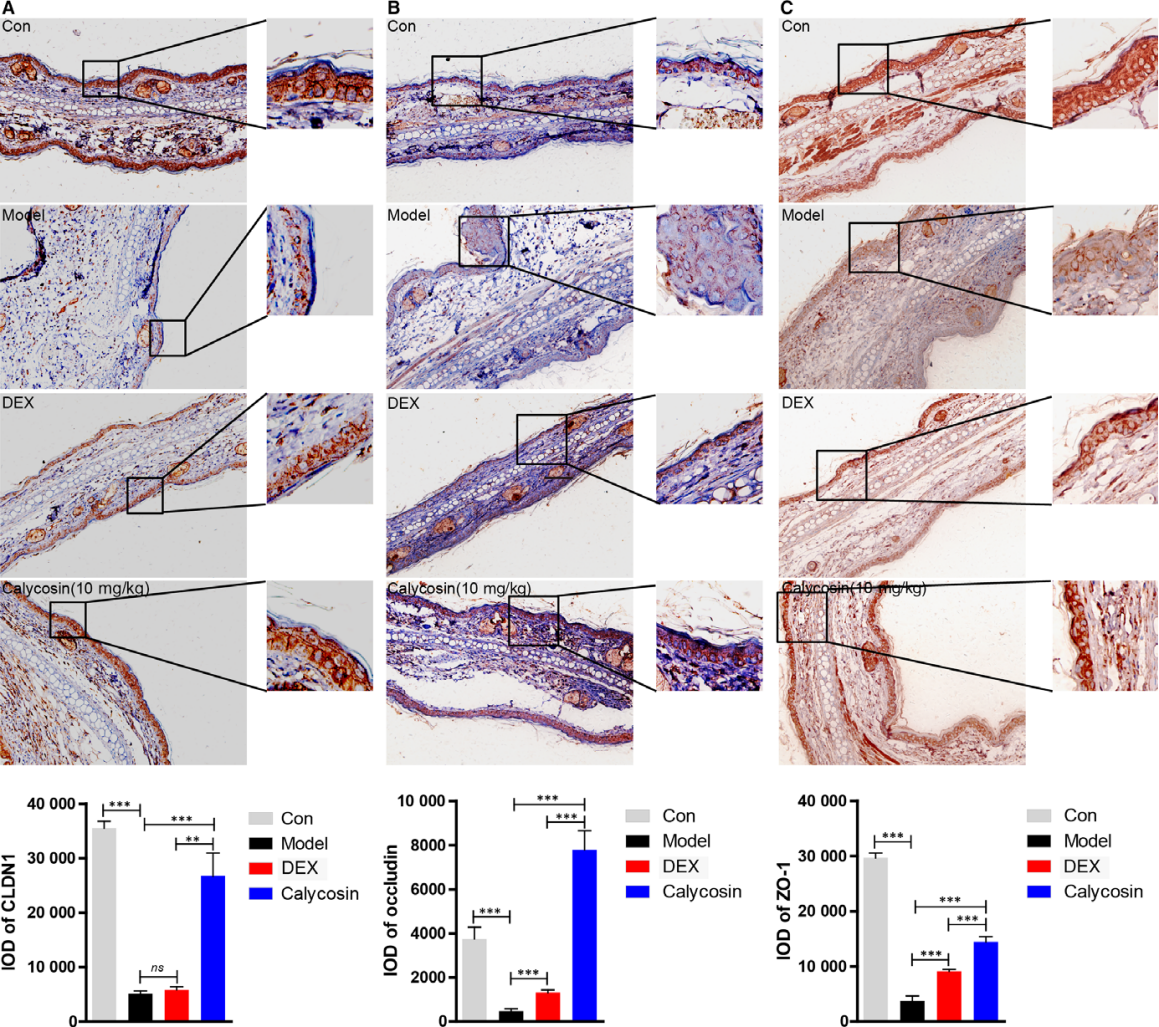
Calycosin improved epithelial tight junctions of ACD mice. On day 7 in the ACD model, mice were killed, and then, the ears were removed and fixed by 10% formalin. A‐C, IHC was performed to detect the expression of CLDN1, occludin and ZO‐1 (n = 5, magnification: ×200). Data of IOD (integral optical density) were counted by IPP (Image‐Pro Plus) software (mean + SD, n = 5, ***P <* *.001, **P <* *.01)

To investigate the effect of calycosin on regulating the TJs, immunohistochemistry was performed to detect the expression of CLDN1, occludin and ZO‐1. The results showed that epithelial tight junctions in ACD model were deteriorated obviously compared with those in control mice, which was characterized by the decrease of CLDN1 (Figure 2A),occludin (Figure 2B) and ZO‐1 (Figure 2C). In contrast to the feebler effect of dexamethasone, calycosin up‐regulated the expression of these tight junctions significantly.

### 3.3 Calycosin repaired epithelial tight junctions in HaCaT cells

**Figure 3:**
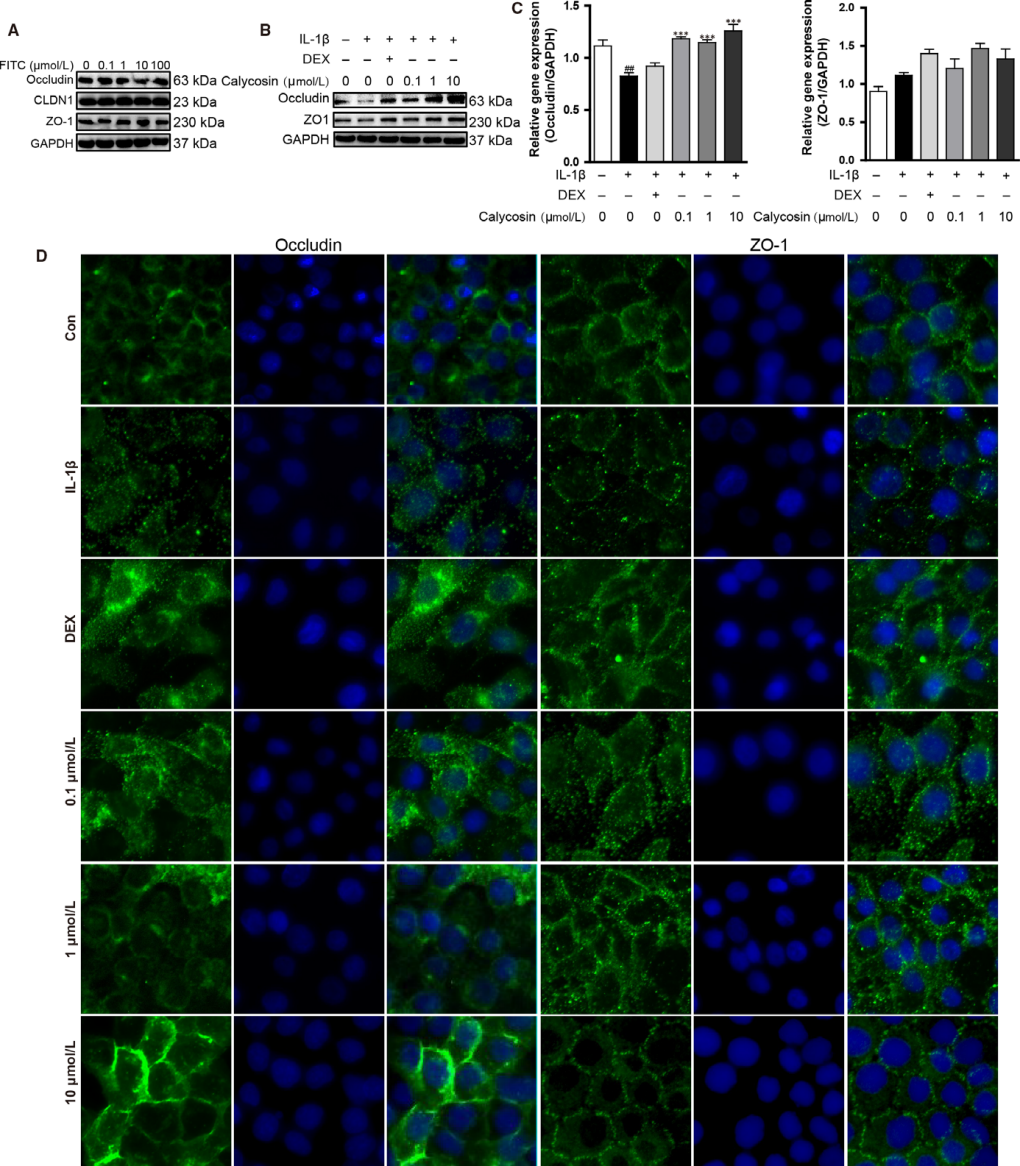
Calycosin repaired IL‐1β‐induced damage of epithelial tight junctions in HaCaT cells. A, HaCaT cells were stimulated with increasing concentrations of FITC (0.1, 1, 10 and 100 μmol/L) for 24 h, then the total proteins were extracted, and Western blot was performed to detect the expressions of occludin, CLDN1 and ZO‐1. B, HaCaT cells were pre‐treated with different concentrations of calycosin and DEX (10 μmol/L) for 6 h and then costimulated with 30 ng/mL IL‐1β for 24 h. Effects of calycosin on protein levels of occludin and ZO‐1 in HaCaT were detected by Western blot. C, The relative mRNA levels of occludin and ZO‐1 were analysed after costimulated with IL‐1β (30 ng/mL) for 6 h (mean + SD, n = 6, ^##^ *P *<* *.01 vs control; ***P <* *.001 vs IL‐1β). D, Immunofluorescence assays were performed to confirm the repairment of epithelial tight junctions by calycosin in HaCaT cells (n = 3; magnification: ×400). The data are representatives of 3 independent experiments

First, we investigated the effect of FITC on tight junctions in HaCaT cells. Increasing concentrations of FITC (0.1, 1, 10 and 100 μmol/L) exposure showed no significant effects on the protein expressions of tight junctions (Figure 3A). Recently, a range of cytokines have been shown to influence tight junction function.^35^ IL‐1β is known to cause an increase in intestinal epithelial tight junction permeability.^28^, ^36^ Accordingly, we determined the effect of calycosin on tight junctions dysfunction induced by IL‐1β in HaCaT cells. Pre‐treatment with calycosin (10 μmol/L) reversed the low expression of occludin induced by IL‐1β (Figure 3B), and the results of mRNA showed similar trends (Figure 3C). Immunofluorescence assay further indicated that calycosin improved expression and distribution of occludin and ZO‐1 (Figure 3D), despite no detectable change of ZO‐1 in protein amount and mRNA expression.

### 3.4 Calycosin reduced the expression of HIF‐1α both in vivo and in vitro

**Figure 4:**
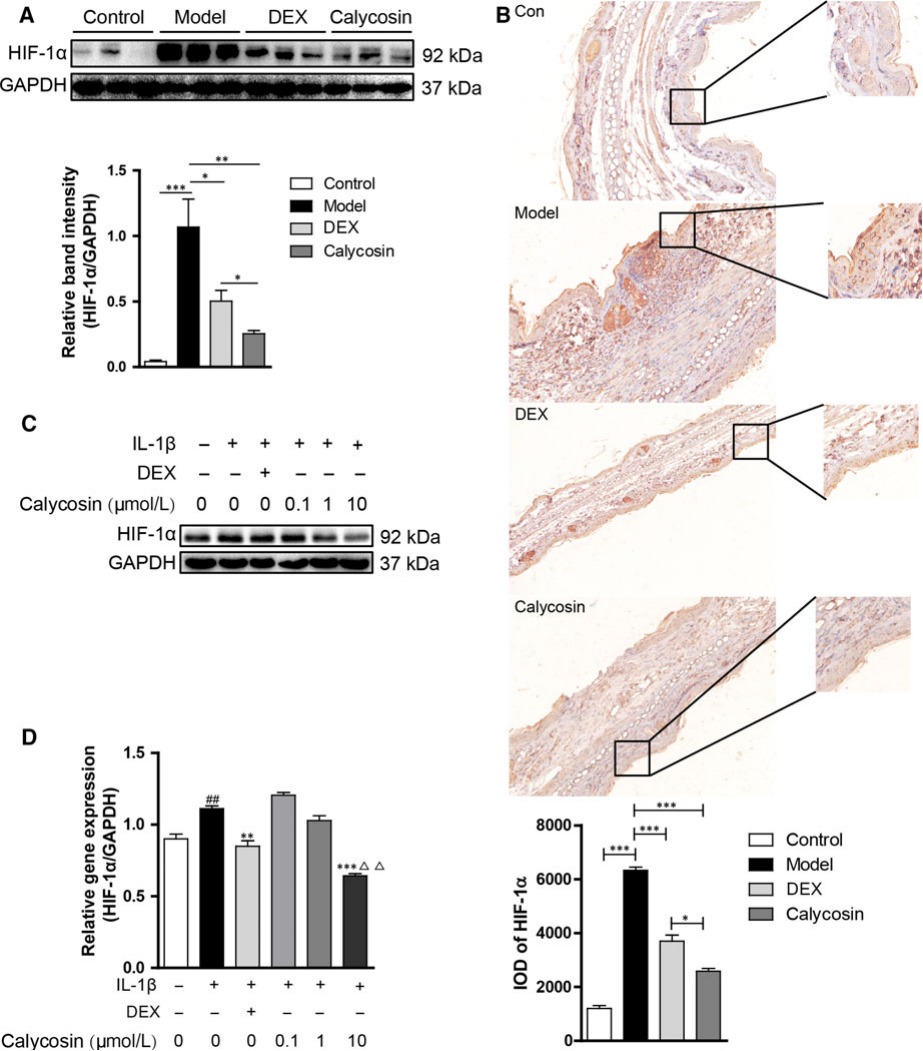
Calycosin reduced HIF‐1α expression in ACD mice model and in HaCaT cells. A, On day 7 in the ACD model, mice were killed, and then, the ears were removed. The expression of HIF‐1α in the total protein was detected by Western blot. The data are expressed as the mean + SD of the ratios of indicated protein to GAPDH, n = 6, ***P <* *.001, **P <* *.01, *P <* *.05. B, IHC was performed to detect the expression of HIF‐1α. C, HaCaT cells were pre‐treated with different concentrations of calycosin and DEX for 6 h and then costimulated with 30 ng/mL IL‐1β for 24 h. Effects of calycosin on protein levels of HIF‐1α in HaCaT cells were detected by Western blot. D, HaCaT cells were pre‐treated with different concentrations of calycosin and DEX for 6 h and then costimulated with 30 ng/mL IL‐1β for 6 h. Effects of calycosin on mRNA levels of HIF‐1α in HaCaT cells were detected by real‐time PCR. ^##^ *P *<* *.01 vs control, ***P <* *.001, **P <* *.01 vs IL‐1β, ^▵▵^ *P *<* *.01 vs DEX. The data are representatives of 3 independent experiments

To address the effect of calycosin on the expression of HIF‐1α, Western blot,immunohistochemistry and real‐time PCR were performed. The results showed that HIF‐1α increased significantly in the ACD model compared with that in control, and calycosin (10 mg/kg) reduced the expression of HIF‐1α obviously (Figure 4A). Immunohistochemistry results also showed that the expression of HIF‐1α was reduced by calycosin (10 mg/kg) (Figure 4B). Furthermore, calycosin (10 μmol/L) could significantly reduce the protein and mRNA expression of HIF‐1α induced by IL‐1β in HaCaT cells (Figure 4C,D). These results suggested that calycosin down‐regulated the HIF‐1α expression both in vivo and in vitro.

### 3.5 Knocking‐down HIF‐1α reversed the down‐regulation of occludin and the redistribution of CLDN1 and ZO‐1 induced by IL‐1β in HaCaT cells

**Figure 5:**
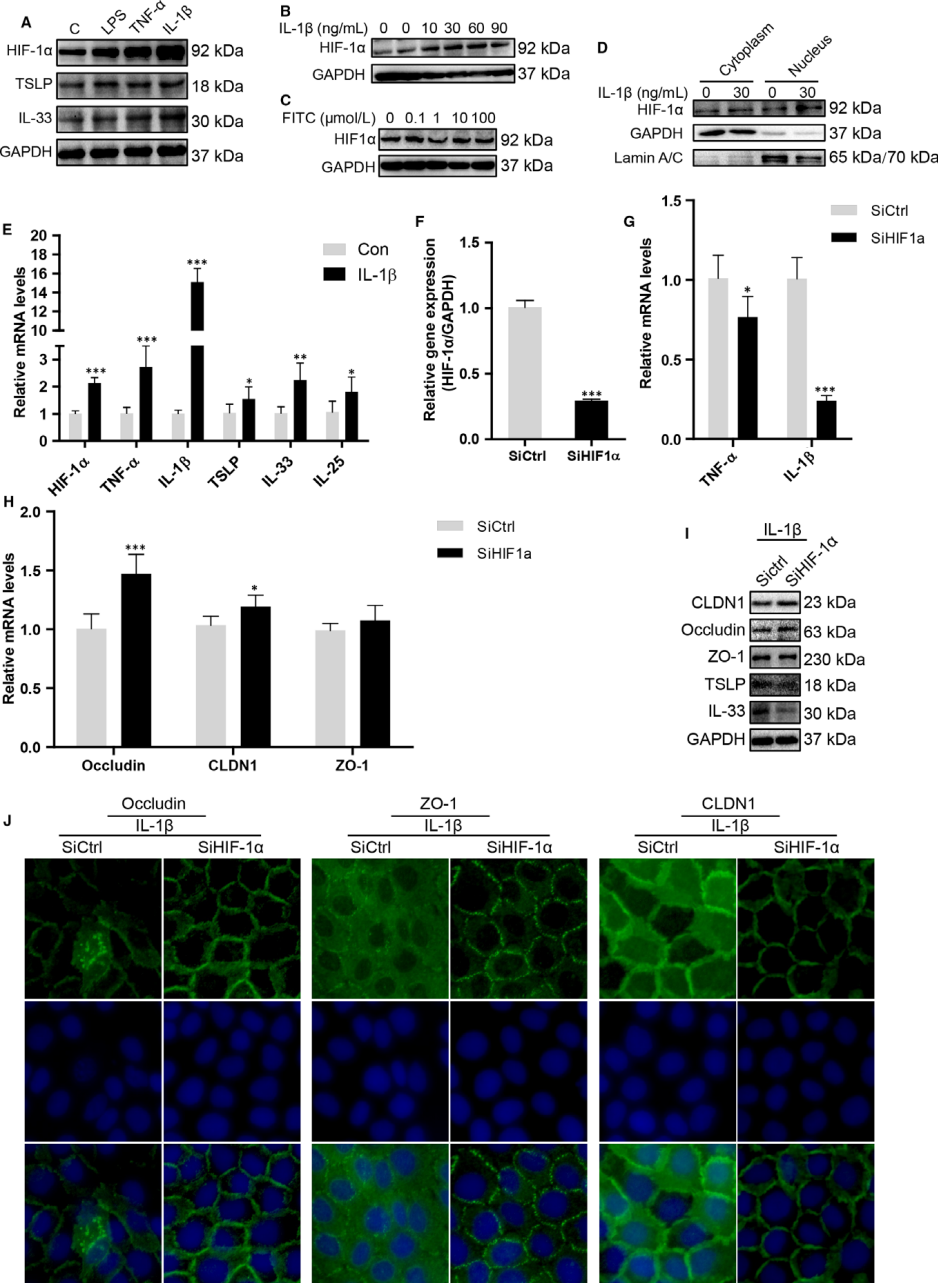
Knocking‐down HIF‐1α reversed the tight junction damage induced by IL‐1β. A, HaCaT cells were, respectively, stimulated with LPS (10 μg/mL), TNF‐α (20 ng/mL) and IL‐1β (30 ng/mL) for 24 h. Proteins in total cell lysates were separated by SDS‐PAGE and immunoblotted with anti‐HIF‐1α, anti‐TSLP, anti‐IL‐33 or anti‐GAPDH, as indicated. B, HaCaT was stimulated, respectively, with increasing concentrations of IL‐1β for 24 h, and the expression of HIF‐1α was detected by Western blot. C, HaCaT cells were stimulated with increasing concentrations of FITC (0.1, 1, 10 and 100 μmol/L) for 24 h, then the total proteins were extracted, and Western blot was performed to detect the expression of HIF‐1α. D, IL‐1β promoted the nuclear translocation of HIF‐1α protein. Nuclear and cytoplasmic fractions were prepared 24 h after IL‐1β (30 ng/mL) stimulation. E, HaCaT cells were stimulated with IL‐1β (30 ng/mL) for 4 h. Then, the relative mRNA expression of HIF‐1α, TNF‐α, IL‐1β, TSLP, IL‐33 and IL‐25 were analysed by real‐time PCR (mean + SD, n = 6, ***P <* *.001, **P <* *.01, *P <* *.05). F‐H, HaCaT cells were transfected with control small interference RNA (siCtrl) or HIF‐1α‐specific siRNA (siHIF‐1α). After 48 h, cells were stimulated with IL‐1β (30 ng/mL) for 4 h. The relative mRNA levels of HIF‐1α, TNF‐α, IL‐1β, occludin, CLDN1 and ZO‐1 were measured by quantitative real‐time RT‐PCR (QRT‐PCR). ***P <* *.001, *P <* *.05. I‐J, HaCaT cells were transfected with control small interference RNA (siCtrl) or HIF‐1α‐specific siRNA. After 48 h, cells were stimulated with IL‐1β (30 ng/mL) for 24 h, and Western blot and immunofluorescence were performed to detect the expression and distribution of CLDN1, occludin and ZO‐1 (n = 3; magnification: × 400). The data are representatives of 3 independent experiments

HaCaT cells were stimulated with LPS, TNF‐α or IL‐1β, respectively, for 24 hours, and the results showed that IL‐1β was the most significant factor in increasing the expression of HIF‐1α (Figure 5A) in a dose‐dependent manner (Figure 5B). However, increasing concentrations of FITC (0.1, 1, 10 and 100 μmol/L) exposure had no detectable change in the protein expressions of HIF‐1α (Figure 5C). The nuclear translocation of HIF‐1α was higher in IL‐1β‐stimulated cells than that in the controls (Figure 5D), which indicates that IL‐1β induces the stabilization of HIF‐1α, leading to its nuclear translocation. Meanwhile, the mRNA levels of HIF‐1α, TNF‐α and IL‐1β were significantly up‐regulated by IL‐1β, and this was accompanied by the up‐regulation of cytokines indicative of allergic disease initiation, including TSLP, IL‐33 and IL‐25 (Figure 5A,E).^37^, ^38^, ^39^

Then, we analysed the effect of HIF‐1α on tight junctions by HIF‐1α gene silence. Compared with that in the control, TNF‐α and IL‐1β expression decreased as a result of HIF‐1α down‐regulation at the mRNA level (Figure 5F,G). Our results suggested that there was a circulation between IL‐1β and HIF‐1α. HIF‐1α may act as an amplifier for inflammatory signal and IL‐1β response, which contributed to the formation of pro‐allergic and pro‐inflammatory microenvironment in HaCaT cells. Given that IL‐1β induced the expression of HIF‐1α, as well as the damage of tight junctions, there may exist link between HIF‐1α and tight junctions. After HIF‐1α was knocked down by transfecting the specific siRNA for 48 hours, the mRNA expressions of occludin and CLDN1 in IL‐1β‐stimulated cells were increased significantly compared with that expressed in control (Figure 5H). Protein expressions of CLDN1, occludin and ZO‐1 were consistent with the results of PCR. Meanwhile, the expressions of TSLP and IL‐33 were down‐regulated by knocking‐down HIF‐1α (Figure 5I). Immunofluorescence assays further showed that HIF‐1α silencing could reverse the deterioration of occludin, ZO‐1 and CLDN1 induced by IL‐1β (Figure 5J). Taken together, these findings suggested that HIF‐1α plays a critical role in the damage of tight junctions.

### 3.6 HIF‐1α inhibitor administered in ACD mice attenuated allergic inflammation and repaired the tight junctions

**Figure 6:**
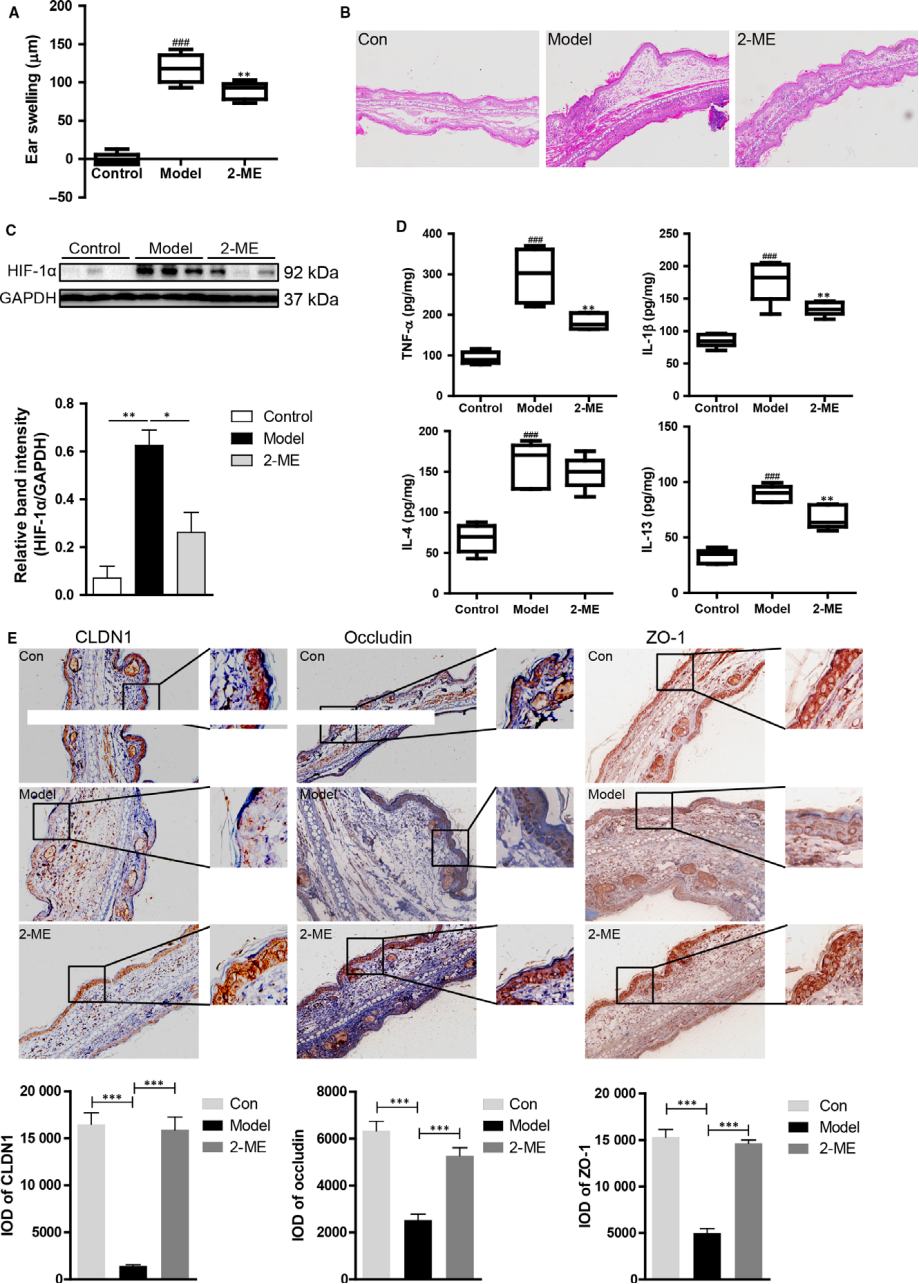
2‐ME inhibited ACD‐associated inflammation and repaired the tight junctions. A, Ear swelling was calculated on day 7 in the ACD mice model (means + SD, n = 6, ^###^ *P *<* *.001 vs control; **P <* *.01 vs model). B, Haematoxylin and eosin (H&E)‐stained ear skin sections from FITC‐induced ACD model mice (n = 5; magnification: ×100). C, The expressions of HIF‐1α in the total protein were detected by Western blot. The data are expressed as the mean + SD of the ratios of indicated protein to GAPDH. **P <* *.01, *P <* *.05. D, The levels of cytokines IL‐4, IL‐13, TNF‐α and IL‐1β in the ear tissue homogenates were detected by ELISA (mean + SD, n = 6, ^###^ *P *<* *.001 vs control; **P <* *.01 vs model). E, IHC was performed to detect the expression and distribution of CLDN1, occludin and ZO‐1 (n = 5, magnification: ×200). Data of IOD (integral optical density) were counted by IPP (Image‐Pro Plus) software (mean + SD, n = 5, ***P <* *.001)

2‐ME, an inhibitor of HIF‐1α,^40^ was administered daily to further determine the effect of HIF‐1α on epithelial tight junction and the follow‐up allergic inflammation. The ear swelling and inflammatory cell infiltration were significantly suppressed by 2‐ME (Figure 6A,B). The levels of TNF‐α, IL‐1β and IL‐13, as well as HIF‐1α, could be remarkably reduced by 2‐ME (Figure 6C,D). 2‐ME could also repair the tight junctions by means of up‐regulating the expression of CLDN1, occludin and ZO‐1 (Figure 6E).

### 3.7 Calycosin repaired the tight junctions by down‐regulating the HIF‐1α expression in HaCaT cells

**Figure 7:**
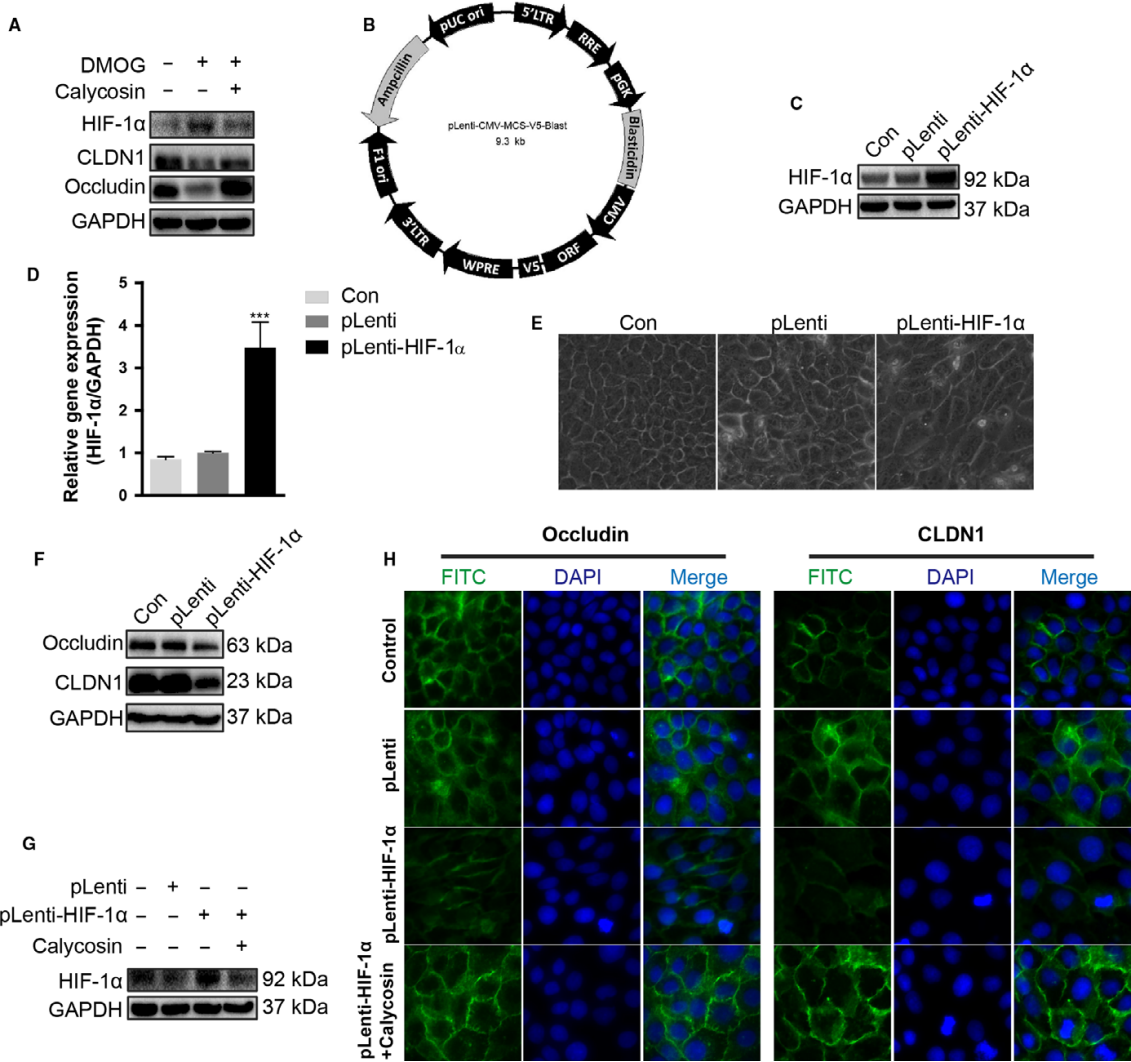
Calycosin repaired the tight junctions by down‐regulating the HIF‐1α. A, HaCaT keratinocytes were treated with DMOG (1 mmol/L) or cotreated with calycosin (10 μmol/L) for 24 h, and then, the expressions of HIF‐1α, CLDN1 and occludin were detected by Western blot. B, The vector map of pLenti‐CMV‐V5‐Blst. C‐D, HaCaT cells, transfected with empty pLenti vectors, or pLenti‐HIF‐1α, were analysed via Western blotting with the specified antibody and real‐time PCR with specified primers (means + SD, n = 6, ***P <* *.001 vs pLenti). E, The morphological changes were observed. F, The expressions of occludin and CLDN1 were detected by Western blot. G, The effect of calycosin on HIF‐1α expression was detected by Western blot after transfected with pLenti‐HIF‐1α. H, The effect of calycosin on tight junctions was detected by immunofluorescence (n = 3; magnification: ×400). The data are representatives of 3 independent experiments

To further testify the role of HIF‐1α in calycosin‐regulated tight junctions, we evaluated the effect of pharmacologic HIF activation by DMOG on the expressions of tight junction proteins. The results showed that DMOG promoted protein accumulation of HIF‐1α and reduced the expressions of CLDN1 and occludin. Calycosin reversed the effect of DMOG on the expressions of HIF‐1α, CLDN1 and occludin (Figure 7A). Furthermore, we overexpressed HIF‐1α in HaCaT cells by transfecting pLenti‐h‐HIF‐1α plasmid (Figure 7B), and transfection efficiency was confirmed by Western blot and qPCR (Figure 7C,D). We found that cell morphology changed after HIF‐1α overexpressed (Figure 7E) and the expressions of occludin and CLDN1 were significantly reduced (Figure 7F). After transfection with HIF‐1α, the expression of HIF‐1α itself was reduced by calycosin significantly. Moreover, the reduced expressions of occludin and CLDN1 could be reversed by calycosin (Figure 7G,H). These results suggested that calycosin repaired the tight junctions by down‐regulating the HIF‐1α.

## 4 DISCUSSION

When allergic dermatitis occurs, it was often accompanied by itching and scratching, which seriously impairs the quality of life. The hallmarks of atopic dermatitis are a chronic, relapsing form of skin inflammation, a disturbance of epidermal barrier function that culminates in dry skin.^16^ Although anti‐inflammatory treatments might reduce the disease severity by suppressing the immune reaction, they do not fully improve the barrier abnormalities that drive disease pathogenesis in AD. Currently, glucocorticoids are the most commonly used drugs for the treatment of allergic dermatitis, but the long‐term application of glucocorticoid often causes serious side effects and it cannot alleviate the recurrence of the disease.^41^

Previously, by cell chromatography, our group identified that calycosin can be effectively combined with 16HBE, an airway epithelial cell line.^12^ In this study, the results showed its effect on repairing epithelial barrier, especially improving the expressions of tight junction proteins such as occludin, ZO‐1 or CLDN1 either in vitro and in vivo. This result might indicate that repairing the tight junctions can relieve the symptoms and episodes of allergic dermatitis. Recent studies also suggest that prevention of AD can be achieved by early interventions protecting the skin barrier.^42^ When exposed to allergens again, repairing the epithelial barrier may become a new strategy for the treatment of allergic dermatitis.^43^ Measures directed at protecting the skin barrier are essential in the treatment of patients with AD, and early intervention may improve outcomes for both the skin disease and other target organs.

Considering the mechanism of calycosin repairing the tight junctions, we investigated HIF‐1α. It has been reported that calycosin reduced NF‐κB activation in LPS‐stimulated human keratinocytes 44 and LPS induced HIF‐1α protein accumulation by activating NF‐κB.^45^ Moreover, the role of HIF‐1α in allergic diseases has been increasingly recognized.^46^, ^47^ A previous study showed that HIF‐1α increases epithelial permeability in human nasal epithelium.^48^ Moreover, tight junction proteins are indicated as MMP (matrix metalloproteinases) substrates. Both occludin and the scaffolding protein ZO‐1 are the substrates for gelatinases, and HIF‐1α can trigger MMP activation.^49^ We considered that HIF‐1α regulated tight junction proteins. In our study, by silencing or overexpressing HIF‐1α in vitro,we demonstrated that HIF‐1α plays a critical role in regulating the inflammation microenvironment and tight junctions. Also, we found that HIF‐1α could be significantly reduced by calycosin both in ACD mice model and in HaCaT cells. Moreover, improvement of occludin and CLDN1 by calycosin was accompanied by the decrease of HIF‐1α in HaCaT cells overexpressing HIF‐1α. These results indicate that calycosin plays a barrier‐repairing effect by down‐regulating the expression of HIF‐1α. We also observed that pLenti‐HIF‐1α‐transfected cells were elongated and larger than control cells, consistent with the morphology of myofibroblasts which normally lack tight junctions,^50^ whereas both parental and pLenti‐transfected cells showed a typical cuboidal epithelial shape. This implied that HIF‐1α might change the polarity of epithelial cells, which was also reported in other researches.^51^

To investigate how HIF‐1α is up‐regulated in allergic dermatitis, we first examined the effects of FITC exposure on the expression of HIF‐1α in HaCaT cells. The results exhibited that FITC exposure showed no significant effects on the HIF‐1α expression. Then, the same trend has emerged in the detection of tight junction proteins. FITC may not perform direct effect on regulating the TJs and the expression of HIF‐1α. It has been reported that after FITC painting, DC becomes rapidly activated and starts their migration to skin‐draining lymph nodes where they initiate antigen‐specific T‐cell responses and then together with pro‐inflammatory signals trigger the functional maturation of DC, which now also up‐regulate expression of costimulatory molecules and, importantly, pro‐inflammatory cytokines in the skin, such as IL‐1α, IL‐1β and TNF‐α. Together, these mediate the activation and proliferation of naïve antigen‐specific T cells as well as their polarization towards appropriate T helper (Th) type 1, Th2 or Th17 effector cells.^52^, ^53^, ^54^ Given that HIF‐1α can be activated by these inflammatory mediators, and can activate inflammation‐modulating proteins itself,^29^ we think that in FITC‐induced ACD model, FITC painting activate DC and finally triggered Th2 immune responses, and in this process, rapid inflammation occurred and many cytokines produced, which then up‐regulated the expression of HIF‐1α and disturbed the epithelial TJs, and disturbed TJs exacerbated allergic inflammation.

To investigate the mechanisms of how calycosin regulates the expression of HIF‐1α, we first considered the proteasome pathway. When oxygen is available, PHDs (prolyl hydroxylases) are active and hydroxylate HIF‐α, marking it for proteasomal degradation in a process mediated by von Hippel‐Lindau tumour suppressor protein (VHL)‐dependent ubiquitination. If oxygen concentration drops, PHDs become inactive, resulting in HIF‐α accumulation.^55^ In our study, we found that DMOG, a small‐molecule dimethyloxalylglycine which inhibited all PHD isoforms,^56^ promoted protein accumulation of HIF‐1α and reduced the expressions of CLDN1 and occludin in HaCaT cells, and calycosin reversed the effects induced by DMOG. Furthermore, by overexpressing HIF‐1α in HaCaT cells, we found that calycosin also reduced the expression of HIF‐1α. These results suggested that calycosin may down‐regulate the expression of HIF‐1α by post‐transcriptional regulation, such as by increasing the efficient PHD activity. In addition, our research in vitro focuses the regulation of calycosin on HIF‐1α expression under normoxia. HIF‐1α stabilization in immune cells can occur in an oxygen‐independent manner by activating NF‐κB.^29^ UVB induces HIF‐1α expression via the JNK, ERK and P38 pathways in human keratinocytes.^31^ Calycosin reduced NF‐κB activation in TNF‐α‐stimulated 16HBE cells or LPS‐stimulated human keratinocytes.^12^, ^44^ Calycosin inhibits the phosphorylation of ERK 1/2 and NF‐κB.^7^, ^57^ Hence, calycosin may also regulate the expression of HIF‐1α by transcriptional regulation.

Our results in vitro also showed that the knockdown of HIF‐1α in HaCaT cells can reverse the levels of TSLP and IL‐33 induced by IL‐1β. Some researches have shown that HIF‐1α is associated with TSLP and IL‐33. In UVB‐mediated immune response in keratinocytes, UVB exposure may increase TSLP expression through a HIF‐1α‐dependent mechanism.^31^ It has been reported that epithelial barrier dysfunction can lead to loss of adhesion between cells and cause the release of allergy‐related cytokines, such as TSLP, in skin keratinocytes.^58^ Recent research by our group showed that CLDN1 down‐regulation in HaCaT cells could exacerbate the production of TSLP.^59^ Calycosin may reduce the production of TSLP and IL‐33 by repairing the tight junctions.

To conclude, calycosin may be a potential drug for the treatment of allergic dermatitis by regulating tight junctions via inhibiting the expression of HIF‐1α, and as a consequence, HIF‐1α and TJs might be possible therapy targets for allergic dermatitis. In addition, this study also suggests the theoretical possibility that the combined treatment of dexamethasone and calycosin might be a useful modality for allergic dermatitis treatment, and further studies involving the combined treatment of steroid and barrier repair therapies are needed.

## CONFLICT OF INTEREST

The authors declare no conflict of interest.
