# Supplementary material for: Calycosin alleviates allergic contact dermatitis by repairing epithelial tight junctions via down‐regulating HIF‐1α
Source: J Cell Mol Med. 2018 Jul 11;22(9):4507–21. doi: 10.1111/jcmm.13763 (PMC6111858; doi:10.1111/jcmm.13763)
Supplement: Supplementary file 1 [file JCMM-22-4507-s001.doc]

**Calycosin alleviates allergic contact dermatitis by repairing epithelial tight junctions via downregulating HIF-1α**

**Supporting Information**


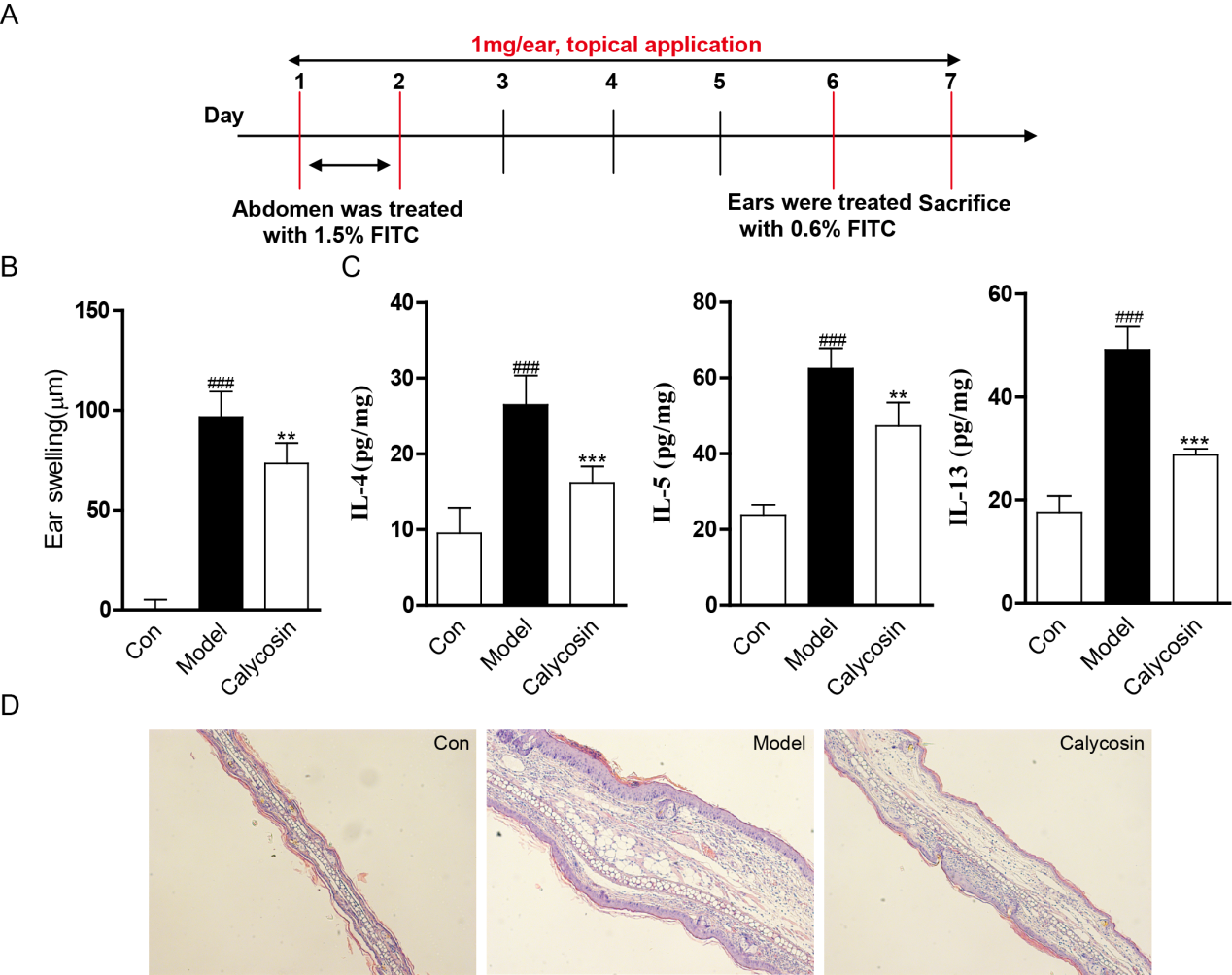


**Fig. S1** The topical application of calycosin alleviates ACD**. (A),** Flow charts of the FITC-induced ACD model and 1mg of calycosin was painted to the ears once daily. **(B)**, Ear swelling was calculated on day 7 in the ACD model mice (means + SD, n = 6, ^###^*P*< 0.001 vs control; ***P*< 0.01 vs model). **(C),** Calycosin reduced the levels of IL-4, IL-5 and IL-13 in the ear tissue homogenates (mean + SD, n = 6, ^###^*P*< 0.001 vs control; ****P*< 0.001, ***P*< 0.01 vs model). **(D),** Hematoxylin and eosin (H&E)-stained ear skin sections from FITC-induced ACD model mice (n = 3; magnification: × 200).


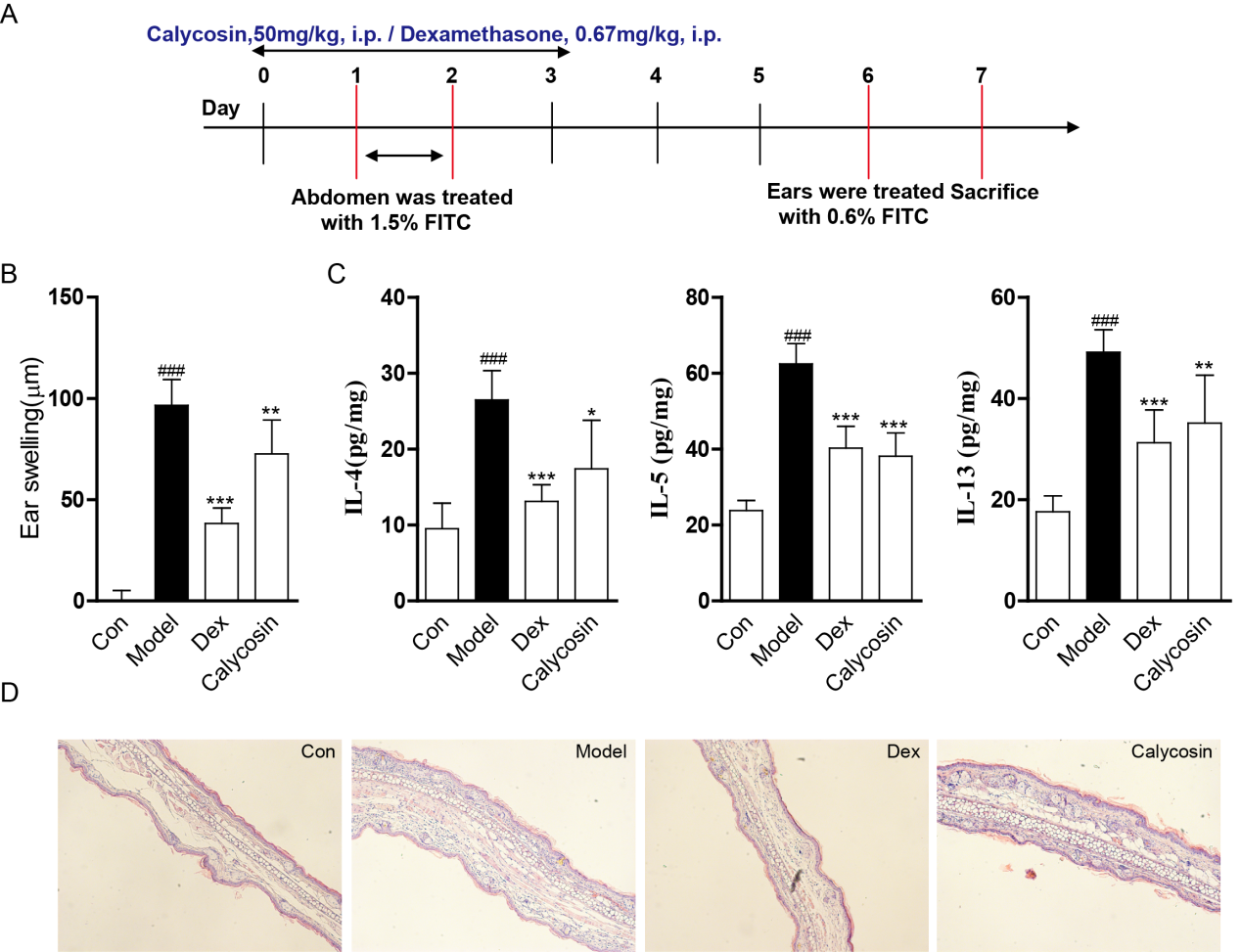


**Fig. S2** Administration of calycosin only in the initial stage of ACD model alleviates allergic inflammation. **(A),** Mice were administered once daily with calycosin (50 mg/kg, intraperitoneally), DEX(0.67 mg/kg, intraperitoneally) or normal saline (model group) 1 day before treatment with FITC until day 3 of the model**. (B)**, Ear swelling was calculated on day 7 in the ACD model mice (means + SD, n = 6, ^###^*P*< 0.001 vs control; ****P*< 0.001, ***P*<0.01 vs model). **(C),** The levels of IL-4, IL-5 and IL-13 in the ear tissue homogenates were reduced by calycosin (mean + SD, n = 6, ^###^*P*< 0.001 vs control; ****P*< 0.001, ***P*< 0.01, **P*< 0.05 vs model). **(D),** Hematoxylin and eosin (H&E)-stained ear skin sections from FITC-induced ACD model mice (n = 3; magnification: × 200).
